# Stakeholder perspectives on veterinary student preparedness for workplace clinical training – a qualitative study

**DOI:** 10.1186/s12917-022-03439-6

**Published:** 2022-09-09

**Authors:** Jennifer Routh, Sharmini Julita Paramasivam, Peter Cockcroft, Vishna Devi Nadarajah, Kamalan Jeevaratnam

**Affiliations:** 1 School of Veterinary Medicine, University of Surrey, Guildford, UK; 2 Division of Human Biology, School of Medicine and IMU Centre for Education, International Medical University, Kuala Lumpur, Malaysia

**Keywords:** Workplace learning, Clinical placements, Rotations, Expectations, Readiness, Preparedness

## Abstract

**Background:**

The success of workplace clinical training (WCT) is important given that veterinary students are licensed to work independently upon graduation. Considering this, it is perhaps surprising that there is limited published work describing what it means to be prepared for this educational experience, particularly given that the transition to WCT can be stressful for students. This paper reports the results of a qualitative study aiming to generate a rich understanding of veterinary student preparedness for WCT using emic, or insider, perspectives of key stakeholders.

**Methods:**

From a constructivist standpoint, homogenous online group interviews were held with final year veterinary students, recent student alumni, clinical supervisors, faculty, and academic educationalists to discuss what it means to be prepared for WCT. The data was analysed using a template analysis approach.

**Results:**

A three-tier taxonomy to describe preparedness for WCT was constructed from the data. At the topmost level, there were seven themes to illuminate different aspects of preparedness: students should be prepared 1) for the transition to learning and working in a clinical and professional environment, 2) for self-directed and experiential learning whilst working, 3) with a growth mindset, 4) with intrinsic motivation and enthusiasm for learning and working, 5) for communication, consultation and clinical reasoning, 6) with the knowledge for work, and 7) with the practical competence and confidence for work.

**Conclusions:**

This study provides a deeper understanding of the tools we can provide, and the attributes we can nurture in, senior veterinary students to facilitate their learning and working during WCT. This improved understanding is a necessary precursor to refining pedagogical support and curriculum design within veterinary schools.

**Supplementary Information:**

The online version contains supplementary material available at 10.1186/s12917-022-03439-6.

## Background

Starting to learn from real patients in the workplace can be motivating for clinical students [1] and it provides them with a focus and an opportunity for growth [2]. However, transitions in clinical education are stressful [1, 3, 4] and they have the potential to impact negatively on learning gain if students aren’t prepared appropriately to learn and work in their new environment [3, 5]. Veterinary curricula are transparent about the competences expected of students, especially upon graduation, but there is no agreed evidence-based guidance on which competences are required for the point of entry into training in the workplace. Since learning occurs by the ‘construction’ of knowledge and skills on top of what students already possess [6] and there is evidence of knowledge ‘re-contextualisation’ when entering the workplace [7], teaching and learning in the early years should be aligned with upcoming workplace learning goals. Gaining an understanding of what foundational tools are required will facilitate curriculum mapping to improve preparedness, should enhance the student experience, and to help to ease the transition to training in the workplace [8].

Workplace clinical training (WCT) constitutes the final component of the undergraduate veterinary school curriculum. It can be understood from a socio-cultural perspective [9] and situated learning theory; students learn how to practise veterinary medicine by ‘legitimate peripheral participation’ in ‘communities of practice’ [10]. Alongside situated learning, which incorporates the informal (opportunistic) and hidden (unintended and inexplicit) curriculum [11, 12], there are also opportunities for more formal coaching and mentoring from clinical supervisors, and expectations of self-directed learning. It is imperative that through WCT students further develop the competences required for safe and independent practice; it is important that WCT is a successful educational experience.

While a smooth journey to becoming a competent veterinary surgeon is desirable, the lived experience is that it is a road punctuated with stepwise transition periods [13, 14]. The factors impacting the transition from veterinary student to practitioner upon graduation have been extensively studied, including in terms of graduate preparedness [15–30]. However, a review of the literature indicates there is exceptionally little published work focusing on the transition into WCT [31]. Additionally, to our knowledge there has been no work on the smaller and more frequent transition points between workplaces within that period.

It is important here to understand educational transitions because the way in which they are perceived offers an insight into the merits and shortcomings of the education system in preparing students for them. There is strong evidence of high student anxiety and stress about the equivalent transition in medical education [1, 3, 4] where, in students’ opinion, it is too abrupt [5]. Parallels can be drawn in veterinary education, although research around this transition is very limited. Dilly and colleagues [32] demonstrated WCT-related stress in veterinary students as greater than occupational stress in the normal population. Reisbig and colleagues [33] also demonstrated that those veterinary students reporting transitional stress are more likely to report a high level of anxiety and depression, and lower levels of life satisfaction and general health. Additionally, performance pressure, excessive stress, and a high workload present learning obstacles for acquiring practical competencies during WCT [32]. In short, comparative studies point to the transition to WCT being difficult and problematic for veterinary students, and struggling with this transition is often attributed to students being ‘under-prepared’ [34].

Preparedness is not a neutral cognitive construct, and its operationalisation can vary. In other words, it can mean different things to different people [35, 36]. In general terms, preparedness can be considered as “an adaptive goal state of readiness to respond to uncertainty” [37] (pg.64); it is concerned with having the tools to manage upcoming but unknown experiences. In this study, preparedness is defined as a measure of the likelihood that veterinary students are going to be competent to learn and work at the expected level during WCT [35].

**Fig. 1:**
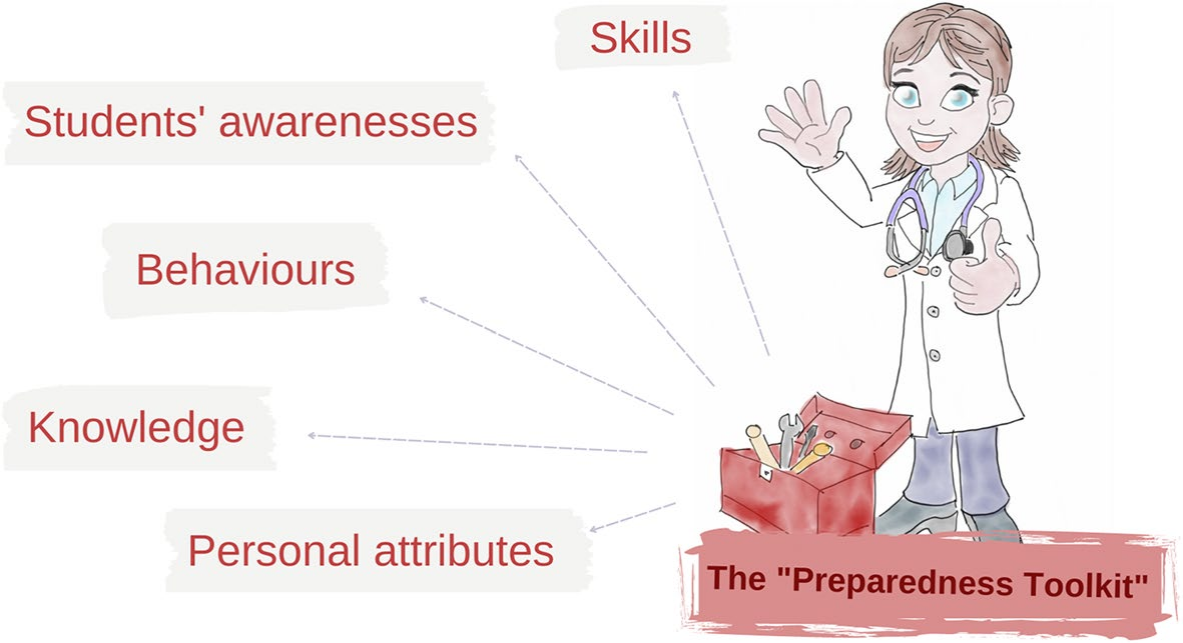
The “Preparedness Toolkit” [42] contains tools that might facilitate preparedness for WCT

However, it is important to acknowledge that the burden of WCT preparation cannot rest solely with students and their pre-clinical educators. This is because students’ competence during WCT is likely to be highly contextualised [38]. In other words, although pre-existing conceptual, procedural, and dispositional knowledge [39] are likely to be important for performance, competence is also fundamentally affected by the activity, organisational practices and workplace culture [40]. Preparedness can also fluctuate over time [41]. For these reasons, some scholars propose that it is not possible to be fully prepared for a transition in clinical education, and it should be viewed as a ‘critically intensive learning period’ (CILP) [40]. Acknowledging this school of thought, the “Preparedness Toolkit” [42] is a useful way to think about preparedness for WCT (Fig. 1). It is a conceptual framework derived from a narrative review of learning theory and contains tools, which if possessed by or nurtured in the student, will assist them in learning and working at the expected level during WCT, whilst negotiating the CILP.

A small study by Saadeh and colleagues [43] has been performed to explore veterinary students’ perspectives of preparedness for WCT using an ‘off the shelf’ quantitative instrument developed in the human health profession education field [44]. The study covered six preparedness themes that were measured in terms of importance. All were rated as important or very important but willingness, and communication and interaction were rated most highly by students. Knowledge and understanding were deemed the least important. Using an instrument developed elsewhere meant that the questionnaire items were not veterinary specific and there were some notable omissions, for example, personal attributes such as animal advocacy. The work also did not address the perspectives of all key stakeholders, particularly those of clinical supervisors or current WCT students.

The aim of this study was to describe the emic perspectives of stakeholders in the United Kingdom (UK) on student preparedness for veterinary WCT. Gaining an emic perspective (the ‘insider view’ [45]) underpins qualitative research which has the intention of evidencing population- and culture-specific insights.

## Results

Forty-four participants took part in fifteen group interviews, representing five UK veterinary schools. In total, there were fifteen clinical supervisors, ten veterinary school faculty, ten academic educationalists, four recent alumni and five current final year veterinary students. Small animal, equine and farm animal practice, and first opinion and referral veterinary practice were all represented.

Table 1 outlines the three-tier taxonomy describing student preparedness for WCT, constructed by the qualitative analysis. Codes represent the most granular way to label aspects of preparedness in the data, which have been subsumed into assembled subthemes and main themes by the researcher. The seven themes of veterinary student preparedness for WCT are expanded forthwith.

**Table 1:** A three-tier taxonomy to describe preparedness for veterinary WCT derived from group interviews with stakeholder groups

Main theme	Subtheme	Preparedness characteristic (code)
**1. Prepared for the transition to learning and working in a clinical and professional environment**	1.1 Aligning expectations of clinical practice with the reality	1.1.1 Students’ awareness of the challenges and realities of practice for veterinary practitioners
		1.1.2 Students’ awareness of the complex professional and cultural norms of the veterinary workplace
		1.1.3 Students’ awareness that their supervisor holds two roles (veterinary surgeon and teacher), and these sometimes compete with each other
		1.1.4 Students’ awareness of the commercial aspects of veterinary practice
		1.1.5 Students’ awareness of the challenges associated with moving workplace clinical training location frequently
		1.1.6 Students’ awareness that there is variation between different workplaces
		1.1.7 Flexibility and adaptability
	1.2 Social and situational awareness	1.2.1 Observing what’s going on in the workplace; an attentiveness
		1.2.2 Teamwork, students putting themselves forward or offering to help
		1.2.3 Social awareness, socially perceptive
		1.2.4 Diplomacy
	1.3 Roles and responsibilities	1.3.1 Students’ awareness of their own roles and responsibilities
		1.3.2 Honesty, integrity, dependability
		1.3.3 Maturity
		1.3.4 Attention to detail
		1.3.5 Calmness, level-headedness
	1.4 Time management	1.4.1 Timeliness
		1.4.2 Personal leadership over breaks and work patterns
		1.4.3 Commitment to completion of tasks
	1.5 Practical aspects—equipment, transport and clothing	1.5.1 Well-presented, wearing appropriate clothing
		1.5.2 Bringing the correct equipment
		1.5.3 Familiarity with the environment, e.g., where things are, how the computer system works
		1.5.4 Able to get to and from workplace clinical training independently
	1.6 Code of Professional Conduct	1.6.1 Students’ awareness of the content of their licensing body’s code of conduct (e.g. RCVS Code of Professional Conduct, AVMA Principles of Veterinary Medical Ethics)
		1.6.2 Appropriate use of mobile phones and the internet
**2. Prepared for self-directed and experiential learning whilst working**	2.1 An awareness that learning in the workplace should be an active and experiential process	2.1.1 Students’ awareness of how they learn during workplace clinical training; an active experiential process
		2.1.2 Students’ awareness of the variation in the caseload that they experience
		2.1.3 Students’ awareness of the value of the entire veterinary team and how students can learn from all of them
	2.2 Finding and filling knowledge gaps	2.2.1 Identifying knowledge gaps and saying “I don’t know that”
		2.2.2 Filling knowledge gaps, self-directed learning
		2.2.3 Asking for help
	2.3 How to gain opportunities for learning in the workplace	2.3.1 Students’ awareness that being proactive, enthusiastic, demonstrating competence, and confidence can bring them opportunities in the workplace
		2.3.2 Proactive in seeking personal learning opportunities
		2.3.3. Asking appropriate questions
	2.4 Prepared for learning and working with peers	2.4.1 Working and learning with other students effectively
	2.5 Administration for learning through work	2.5.1 Reading the preparation material provided
		2.5.2 Self-discipline and organisation
		2.5.3 Setting reasonable personal learning objectives
		2.5.4 Students’ awareness of their expected learning outcomes (set by veterinary school or licensing body)
**3. Prepared with a growth mindset**	3.1 Learning to fail and failing in order to learn	3.1.1 Students’ awareness that perfection is not expected; failure or mistakes are likely, and they are part of the learning process
		3.1.2 Resilience in the face of failure, low-level stress and the pressure of the workplace
	3.2 Engaging with feedback	3.2.1 Seeks feedback
		3.2.2 Receptivity to feedback, including critical or constructive feedback
		3.2.3 Understanding what both formal and informal feedback looks like in the workplace
	3.3 Reflection skills	3.3.1 Engaging in meaningful reflection
		3.3.2 Self-awareness of limitations, strengths and weaknesses
		3.3.3 Appropriate level of self-confidence
		3.3.4 Students’ awareness of their own and others’ mental wellbeing, and the importance of self-care
**4. Prepared with intrinsic motivation and enthusiasm for learning and working**	4.1 Motivation	4.1.1 Motivated to learn for a career in veterinary medicine, not for a grade or as a tick box exercise
		4.1.2 Animal advocate
	4.2 Enthusiasm	4.2.1 Enthusiasm
		4.2.2 Willing to try new practical skills with appropriate support
	4.3 Appreciating transferability	4.3.1 Open to learning about species not of particular career interest
		4.3.2 Students’ awareness of the transferability of skills learned during workplace clinical training
**5. Prepared for communication, consultation, and clinical reasoning**	5.1 Communicating with the clinical team	5.1.1 Team communication skills
		5.1.2 Listening to the clinical supervisor
		5.1.3 Polite, respectful
		5.1.4 Personable and friendly
	5.2 Communicating with clients	5.2.1 Client communication skills – able to deliver and discuss information
		5.2.2 Telephone skills
		5.2.3 Empathy, compassion, kindness
		5.2.4 Able to structure and lead a consultation including history taking
		5.2.5 Listening and reacting with appropriate follow up questions during history taking
		5.2.6 Written communication skills
	5.3 Clinical reasoning for common cases	5.3.1 Having a clinical reasoning framework for common problems
		5.3.2 Able to assimilate and understand the importance of clinical information in the case
		5.3.3 Logical, independent thought processes and making sensible attempts to reason
		5.3.4 Taking into account non-medical, owner or contextual factors during clinical decision making
		5.3.5 Clinical reasoning skills when faced with multiple clinical problems
		5.3.6 Knowledge of common differential diagnoses
		5.3.7 Problem solving and forming problem and/or differential diagnoses lists
		5.3.8 Engaging with evidence based veterinary medicine (EBVM)
		5.3.9 Capable of proposing justified and rational clinical decisions
		5.3.10 Students’ awareness of uncertainty and risk in clinical decision making
		5.3.11 Students’ awareness that there’s more than one way of doing something
**6. Prepared with the knowledge for work**	6.1 The “-ologies”	6.1.1 Appropriate knowledge of anatomy
		6.1.2 Appropriate knowledge of pharmacology and therapeutics
		6.1.3 Appropriate knowledge of physiology
		6.1.4 Appropriate knowledge of animal husbandry and production systems
		6.1.5 Appropriate knowledge of parasitology
		6.1.6 Appropriate knowledge of the core vaccines for the principal domesticated species
	6.2 Clinical application of knowledge	6.2.1 Integrating and applying knowledge to cases
**7. Prepared with the practical competence and confidence for work**	7.1 Knowing how is important	7.1.1 Know how to perform practical skills (and not necessarily be able to perform them)
	7.2 Competence and confidence handling animals	7.2.1 Competence and confidence handling animals
		7.2.2 Working safely
	7.3 Basic clinical skills	7.3.1 Basic clinical skills e.g. blood sampling, placing an intravenous catheter
		7.3.2 Surgical dexterity and tissue handling
		7.3.3 Able to use diagnostic equipment e.g. use a microscope
		7.3.4 Able to use a formulary or product data sheets, and calculate drug doses
	7.4 Clinical examination skills	7.4.1 Clinical/physical examination skills
		7.4.2 Appropriate knowledge of what’s normal on a clinical exam e.g. temperature, pulse and respiration rates

### Prepared for the transition to learning and working in a clinical and professional environment

This theme describes how students can be prepared for the physical transition from learning predominantly in the classroom to working and learning in veterinary practices and hospitals.

It was deemed important that students understand the lived experience of learning and working in the clinical workplace including navigating complex social arenas where the culture varies from place to place. To facilitate this, social and situational perceptiveness were highly regarded. Students should be prepared to observe their environment and take the initiative by being proactive in daily tasks. Consequently, being prepared for teamwork was very highly valued; students should offer to help with all aspects of practice:

*“…just being cognisant of the people that they are working with, and how their tasks and their day are going…be able to see it from other people’s perspectives, and that actually…if the nursing staff are really busy, it’s going to be really, really helpful to clear up after you’ve done something”* R1G13 (clinical supervisor)

Having said this, students were identified as *“a colleague rather than someone there to kind of observe and just learn”* (R1G11, student), and they have responsibilities directed towards patient care. Participants thought that students need to be aware of these responsibilities, to understand the implications of their actions in the clinical setting, be honest and dependable whilst working with attention to detail and maturity.

Practical aspects of preparing for WCT were discussed. These included students ensuring that they can independently arrive on time and leave flexibly, wear suitable clothing, and bring the correct equipment. Some clinical supervisors had particular personal grievances, and one exclaimed: *“It drives me crazy. I’m like: what were you expecting to do on this visit, if you didn’t bring your own stethoscope? Were you going to get involved?”* (R2G12, clinical supervisor). Familiarity with the workplace environment was deemed important and this included knowing where frequently used equipment is, how the staff facilities work and how to use the relevant computer system.

Students’ awareness of the Royal College of Veterinary Surgeon’s Code of Professional Conduct will help to prepare them for WCT, according to participants. Aspects of the Code that were identified as particularly relevant related to the appropriate use of the internet, mobile phones and social media, the prescribing cascade, the importance of written clinical notes and obtaining informed consent.

### Prepared for experiential and self-directed learning whilst working

This theme describes how students can be prepared for new approaches to learning:

*“I think it’s that transition from us imparting knowledge on them to them going out and seeking knowledge, and that’s quite a big jump for them to make when they’re used to lectures and seminars and things, and then suddenly they get dumped in the corner of a consulting room.”* R1G3 (faculty staff)

Students should not necessarily anticipate being taught during WCT. Instead, staff identified that students need to be prepared to actively engage in, and learn from, the undertaking of clinical work. However, students should have “*the expectation of: you’re going to see things but you’re not going to see everything”* (R2G11, student), that clinical supervisors are “*not controlling the caseload*” (R1G3, faculty) and their days won’t be continuously filled with “*fire brigade stuff*” (R1G2, faculty). Several means to afford opportunities for engaging in clinical work were identified by participants: student proactivity and being helpful, engaging with the clinical team and demonstrating enthusiasm, self-confidence, and competence. Looking in the workplace diary ahead of time and asking to shadow supervisors on particular cases were highlighted by several participants.

A key aspect identified in preparing for learning in the workplace is that students have *“an awareness of gaps in [their] knowledge, and in a positive way, not in an “I can’t do that” and then giving up, in an “I don’t know how to do that, therefore, I’m going to do something about it”. That’s a very good student, that can see gaps and fills them.”* (R3G14, clinical supervisor).

Students need to appreciate that they can learn in an experiential manner from all members of the team, particularly the veterinary nurses. One student participant offered some useful insights: *“they (nurses) are the ones that are doing a lot of the inpatient care… I’ve found a lot of times the nurses are a little bit less rushed off their feet than the vets…They’re quite happy to take a step back and show you things… I think a lot of them have experience in clinical training because they have so many student nurses or they’re already mentors themselves”* (R1G11, student). Similarly, a well-prepared student would have a supportive, rather than competitive, approach to working with their peers, understanding that what they can learn from each other is valuable. Employing diplomacy and taking shared responsibility with peers was important for working together to provide patient care.

Despite the experiential and unpredictable nature of WCT, participants spoke about how students need to be prepared with an understanding of their personal and University dictated learning objectives, but whether students were expected to communicate these to the clinical team in advance varied. It was also considered important that students should have prepared for WCT by reading the written information provided to them. This specifies information in terms of what to expect and what is expected of them, in advance.

### Prepared with a growth mindset

This theme describes how students can be prepared by possessing a growth mindset. A growth mindset was defined by one participant as *“using the opportunity to learn and seeing [that] fail just means ‘First Attempt In Learning’ and seeing feedback, and all of that, as being important*” (R4G5, academic educationalist), which aligns with the literature in the field [46].

It was identified as important for students to have realistic expectations of themselves; that they cannot and will not know, or be able to do, everything, and that learning from their failures is very important. Furthermore, an awareness that there is support available for students when they do fail and that it is a primary mechanism for building resilience would prepare students well. Students should *“realise that there’s no magic switch that happens at the end of fourth year before final year that actually, you know, you’ve got to, yourself, realise how you’re going to cope with that and how you’re going to be a little bit resilient and get through rotations.”* (R1G1, academic educationalist). Resilience was expected by participants in the face of assessments and feedback, hard work, low-level stress, and the pressure of the workplace.

The ability for students to be able to engage in the feedback process and view it as a two-way process was universally acknowledged as important for WCT. Participants described that students need to be prepared to separate themselves from critical feedback and not to interpret it as a personal criticism. One member of faculty explained that *“the most important personal attribute is that…you can process it (critical feedback) and actually move forward quickly, and not dwell on it, and not allow it to consume you or to impact on things moving forward”* (R2G3, faculty). Students that seek feedback were viewed as well-prepared, although it was recognised that informal feedback can be challenging to acquire in the moment.

Reflection skills were described as essential for the prepared student, particularly in response to feedback. Participants described that students should be aware of their own strengths and limitations, their mental wellbeing, professional identity development, personality and how they might fit into a team in advance of commencing WCT. Reflection and self-awareness were described as requirements for determining students’ self-confidence, which should be adequate but appropriate. Self-confidence encompassed the confidence to admit not knowing, in the knowledge that the student possesses, to communicate in the workplace and to pursue learning opportunities*.*

### Prepared with intrinsic motivation and enthusiasm for learning and working

This theme describes how students can be prepared with intrinsic motivation which manifests as genuine enthusiasm for WCT. Intrinsic motivation is autonomous, driven by curiosity, interest and an inherent satisfaction in learning [47].

It was described as desirable that students are motivated to learn during WCT by an underlying desire to be a competent veterinary surgeon, stemming from a position of animal advocacy: *“I think for me the most important attribute that trumps all others is having compassion for the patients”* (R2G15, clinical supervisor). This is opposed to fulfilling tick box exercises, passing exams or performing well under the pressure of a supervisors’ probing, although frequently faculty staff and clinical supervisors found this to be the case*: “they definitely have a frustrating mindset of like I’ve passed that exam so I can forget about that information”* (R2G2, faculty). Well-prepared students were described as enthusiastic, curious, and willing to try new things, with appropriate support.

Students’ awareness of the transferability of skills learned in WCT was identified as desirable, and this was expressed as *“being proactive and enthusiastic even if they’re not, it’s not where they see themselves working at the end of it”* (R1G2, faculty). These transferable skills not only sit in the clinical space, but the non-clinical and professional space too, as one clinical supervisor explained: *“I try to push home that yes, you may not want to do farm for the long run, but actually some of these communication skills and time management, that’s the stuff that is going to get you a complaint early doors…You can practise that anywhere. So, I try and push the professional skills thing a bit, as something that’s great to practise.”* (R2G12, clinical supervisor).

Additionally, it was described that if students have gained competency in separate skills, they should understand that they are likely to be able to draw from across their training to perform whole professional activities competently*: “it’s like we’ve given them the tools and they’ve never built a ship before, but they’ve built this and they’ve built that, and so therefore they can probably go and do it.”* (R2G4, faculty). This linked to students’ confidence to ‘have a go’ with appropriate support in place.

### Prepared for communication, consultation, and clinical reasoning

This theme describes how students can be prepared for interacting with clients and the clinical team, and moving towards resolving clinical cases.

Being prepared to communicate with a clinical team was described as a critical student skill for WCT: “*I think one of the most important student things for me is communication…the ones that are communicating effectively with us as a big team are the ones that are doing the best.”* (R4G8, alumnus). Positive non-verbal communication skills, being respectful and personable were highlighted. Some faculty suggested that students should be prepared for dealing with conflict or concerns with their supervisor directly, openly and with maturity.

Case presentation skills for ward rounds were valued by staff; the best was described as *“thorough but concise…and not give you every little detail that’s not important”* (R1G15, clinical supervisor) with accurate terminology appropriate for the target audience. Having opportunities to practice vet-to-vet handovers and communicating clinical information in preparation for WCT were appreciated by current students.

A well-prepared student was described as confident in communicating with clients, this included delivering and discussing clinical information, writing clinical notes, using the telephone, and making small talk: *“some of them will stand and chat the client which I’m always like well impressed with”* (R2G10, clinical supervisor). However, there was some disagreement about whether students should be competent at handling clients in especially difficult situations, such as euthanasia. For some participants it wasn’t necessarily expected as “first day” skill, but other clinical supervisors had more confidence in their students and felt they should be prepared to tackle anything: *“as long as they’re prepared on the content, I think they do have the skills of empathy and dealing with confrontation… They have to be, you know, ready to have a go at these things.*” (R1G13, clinical supervisor).

Students should be prepared to lead a structured consultation including the acquisition of the clinical history for a simple case and gathering the required information for planning the next steps, in a timely manner. They should understand the underlying framework of a consultation but also be prepared to listen, observe and “*adapt according to what they’ve heard or what they’ve seen*” (R1G7, academic educationalist).

Being prepared for clinical reasoning was described as challenging but important. Participants described their expectations that students *“should have the framework for the approach for a pretty standard, simple, common case but so well embedded that you almost don’t have to think about that anymore”* (R2G5, academic educationalist). This included being able to integrate and apply adequate knowledge, analyse clinical information from multiple sources and generate problem or differential diagnoses lists. An ability to verbalise thought processes and the logic underlying decisions was also deemed useful for clinical supervisors: *“if they can say “Well, this is what I’m thinking because of this” then you can say “Well, that’s a really sensible conclusion but in this case have you thought about that?” that enables you to have a really good discussion with them.”* (R4G8, alumnus).

An awareness of some of the nuances of clinical practice: that cases often aren’t “black and white”, that there’s likely to be more than one way to treat a patient, and that there’s uncertainty and risk involved in clinical decision making were identified as important, but supervisors often found students are unprepared for:

*“They do it (clinical reasoning) very well when it’s an artificial case that makes sense…It falls down, what they’re less prepared for…is they hate things that don’t make sense.”* (R2G13, clinical supervisor)

*“Often we are dealing with uncertainty, but although we say that, and try and demonstrate it through the cases, I’m not sure they really get it.”* (R1G13, clinical supervisor)

### Prepared with the knowledge for work

This theme describes how students can be prepared for workplace clinical training with appropriate knowledge. It was interpreted that knowledge was perceived as a less important facet of preparedness by staff, as it was remarked by several participants: *“we’ve not mentioned at all, like, student skills or knowledge”* (R3G2, faculty). Although they did concede that “*knowledge is generally excellent*” (R1G2, faculty) since students have typically just sat their exams when they commence WCT, and it might be perceived as less of a problem as a result. In the same interview, it was commented that *“[knowledge is] much, much better than I would have anticipated…what’s sad is their emphasis on the knowledge*.” (R3G2, faculty). In line with this, student talk focussed on knowledge relatively more.

Anatomy knowledge, particularly topographical anatomy, was attributed importance with respect to its relevance in surgical, diagnostic and clinical skills. Pharmacology was also frequently highlighted as important to know for WCT, in particular the basic drug classes. Staff emphasised the importance of students being able to use a formulary and reason why they made therapeutic choices, whereas students were more focused on building up a memorised bank of common drug names (sometimes including commercial trade names) and dose rates:

*“I really don’t think they need to know every drug…They just need to know some basic groups of medicines and how to look it up.”* (R1G6, clinical supervisor)

*“I think as well as learning the common drugs, also learning the common brand names that go with the drugs. So, you can just slip into the practice and use the right language.”* (R2G9, student)

Three separate groups of staff highlighted frustration at a lack of student knowledge of the core vaccines in the main species. Knowledge of animal husbandry was identified as clinically important by large animal supervisors, but they explained that this was often forgotten by students as the subject is covered relatively early in the course.

### Prepared with the practical competence and confidence for work

This theme describes how students can be practically skilled in preparation for WCT.

Competence and confidence handling patients was the most important practical skill that students can be prepared with according to participants. It contributes to clients’ first impressions, affords the student further learning opportunities such as practising physical examination, and several participants spoke about its importance with respect to safe working: *“one thing I probably expect the student to know is how to safely handle the species you’re working with that’s safe for both you and the client, and the animal.”* (R1G11, student). It was also acknowledged that it is important for students to be prepared to identify their own limits with animal handling skills.

Beyond animal handling, talk of practical skill expectations tended to fall into one of two groups: either 1) it was impossible to commit to any specific benchmarks because “*students are good at different things and different students would have had different opportunities, and actually our job in rotations (WCT) is to level that up”* (R2G1, academic educationalist), or 2) that they aligned with the curriculum of the veterinary school as a lower limit: *“I think OSCEs (observed structured clinical exam) offer at least some sort of benchmark”* (R4G2, faculty). What was perceived to be more important were general psychomotor skills such as competency in handling sharps and drawing up medication, muscle memory and confidence in using surgical instruments, and appropriate tissue manipulation.

Instead of focusing on being able to do, many participants expected students to *“know how to do things”* (R1G14, clinical supervisor). This bolsters students with the confidence to make attempts under supervision if appropriate or if not, it can allow them to still contribute to patient care by preparing for the procedure, for example if they know where to clip the patient, or what equipment to get ready.

A well-prepared student should be able to perform a basic, systematic, and precise clinical examination determining when a thorough “top to tail” exam versus a problem-based exam is indicated. There were varying perspectives on the expected ability of students to detect abnormalities, without a clear staff versus student divide. However, it was universally recognised that students should know the normal numerical parameters on day one of WCT: *“I think they should know normal for like, the numeric things, but I think some of the subtleties…I wouldn’t necessarily expect them to know the difference between. I would by the end of the rotation, but not necessarily on day one.”* (R2G15, clinical supervisor)*.*

## Discussion

This research is, to our knowledge, the first to describe multiple stakeholders’ perspectives of student preparedness for veterinary WCT. By performing a qualitative study using group interviews we have been able to gain a rich and contextualised understanding of this complex phenomenon. This was facilitated by using a conceptual framework developed from a review of learning theory to direct data collection and the initial stages of analysis, whilst also inductively generating codes from a range of stakeholders with real experience of WCT.

### Preparedness for the formal, informal, and hidden curriculum of WCT

What and how students learn during WCT can be, albeit somewhat artificially, divided into the formal, informal, and hidden curriculum [48]. The informal curriculum denotes much of what occurs in the clinical environment; unplanned instruction by anyone who is teaching (clinicians, nurses, even peers), according to what the teacher thinks students should acquire in terms of knowledge, skills and attributes [49]. This is reflected in much of participants’ discussions centered around students being prepared to learn from authentic, variable, and opportunistic experiences in clinical practice (Table 1, item 2.). This included how to secure those experiences (2.3), how to engage with them (2.1), what to know for them (pre-existing subject matter) (6.) and what to know about them (how these experiences work in the real world) (1.1).

Preparedness for the formal component of WCT was talked about comparatively little and was primarily concerned with students being aware of their documented expected learning outcomes (2.5.3, 0.4) and how to engage with formal (summative) feedback (3.2). The formal curriculum includes the planned content of WCT such as tutorials or portfolio production, which were essentially unspoken of in terms of preparedness possibly because they only constitute a small proportion of WCT, and students are likely to have experienced this type of formal education before transitioning to WCT.

Elements of the hidden curriculum were exposed in the interviews. The hidden curriculum reflects the unintended and inexplicit influences on students’ learning; the “underbelly” of practice. It originates from the institution’s organisational structure and incorporates its complex customs, rituals and culture [48]. However, the hidden curriculum is often communicated person-to-person through student/teacher interaction and role-modelling. Magnier and colleagues [31] identified veterinary students’ ability to link seamlessly into the workplace as important for successful WCT. This would require students to have an awareness of “how we do things around here” (1.1), a fundamental facet of the hidden curriculum, and the results of this study explain how students can achieve this. The cultural norms of the workplace (1.1.2) and the tension between evidence-based best practice learned from the formal curriculum versus contextualised reality (1.1.1) were identified as entities that students would benefit from being aware of for veterinary WCT. Both of these concepts have been identified in the medical hidden curriculum [50]. Further examples of how students can be prepared to negotiate the hidden curriculum included how they should be aware of when it might be appropriate to ask questions in stressful situations or in front of clients (1.2.3), that being proactive and helpful can afford subsequent opportunities for authentic experiences (2.3.2), and awareness of when it is, or is not, appropriate to verbalise honest career intentions to members of the clinical team (1.2.3). This affirms that the hidden curriculum, which was previously thought to negate the formal curriculum, can be harnessed in a positive manner and improve student experience when there is increased awareness of it.

### Preparedness for a transition to WCT

A scoping review from Atherley and colleagues [51] demonstrates that research focuses on the transition into the medical analogue of WCT in three different ways. Firstly, as a problem to be addressed using curricular innovations to ‘narrow the gap’ between classroom and workplace learning [52, 53]. In this study, ‘narrowing the gap’ was talked about in terms of preparedness where students have all the knowledge and skills required to smoothly transition into WCT and do not feel overwhelmed. This also included some talk of student orientation and familiarisation with the physical workplace, usually through classroom-to-clinic courses.

Secondly, Atherley and colleagues describe the transition through a sociocultural lens, focusing on how students build relationships and navigate the culture of their new learning environment. The social aspects of the transition were addressed in this study as participants talked about preparedness for forming team, client, and peer relationships in depth (5.1, 5.2, 2.4). In this context, a prepared student can participate legitimately in the community of practice and access learning opportunities.

Thirdly, the transition to WCT can be viewed as an opportunity for personal and professional transformation [40], a critically intensive learning period (CILP), and we should aim to empower students to learn in the workplace using reflection and transferable learning skills [51]. The developmental aspect of the transition to WCT was also spoken about by participants in this study. The importance of reflection and the growth mindset (3.) was emphasised, particularly by staff, and was constructed into its own theme. This is supported by evidence that veterinary students with a growth mindset are less likely to report being anxious about the transition to WCT than those with a fixed mindset, although it is an oversimplification to suggest that those with a growth mindset are not anxious at all [54]. Self-awareness of mental health and wellbeing (3.3.4) was also highlighted, and this is recognised as a requirement for students to be able to cope with a CILP.

### Contributing to the ongoing scholarly conversation around preparedness

The results of this study expand on the findings of a survey-based, single-stakeholder study that willingness, knowledge and understanding, professionalism, interpersonal skills and personal attributes are important in terms of student preparedness for veterinary WCT [43]. There is some published work answering related research questions from which important aspects of student preparation for WCT can be inferred such as animal handling and physical exam skills [31, 55, 56], trustworthiness [56], respect [57, 58], communication [31, 55, 56, 59], wearing the correct uniform [57, 58], time management [57], enthusiasm [31, 56, 58], timeliness [58], decision making [59], intellectual curiosity [57], filling knowledge gaps [31], confidence [59], animal advocacy [56] and teamwork [56]. These aspects of preparedness were recognised as important by participants in the interviews reported here. However this qualitative study has added to the ongoing work in this area by painting a more detailed and richer picture of preparedness in terms of specific student behaviours, awarenesses, personal attributes, skills and knowledge.

Short-term and long-term conceptualisations of preparedness have been described by Ottrey and colleagues [36] in the wider health profession education field and were alluded to by participants in the group interviews reported here. The former are characteristics that are useful in the immediate future i.e., in negotiating the imminent transition into WCT. These included familiarity with the physical workspace (1.5.3) or practical aspects, such as having the correct equipment (1.5.2) or wearing a professional uniform (1.5.1). In contrast, long-term preparedness can be utilised beyond WCT and was value-laden in this study, particularly by staff, who perhaps could appreciate how useful these characteristics would be across a veterinary surgeon’s career. These included having a growth mindset (3.), resilience (3.1.2), self-directed learning (2.2), and communication skills (5.1, 2).

### Methodological considerations and limitations

The Covid-19 pandemic forced the interviews to be held online. A major benefit was that data collection could include geographically distant stakeholders in various roles, across several institutions, and this is likely to have enhanced the data richness and breadth as a result. Sampling from a broader population improves the ecological validity [60] and horizontal generalisability [61, 62] of the research. However, the pandemic has had an impact on the content of the data. Given its prominence in the collective psyche at the time of data collection, it is unsurprising that there was a keenness to talk about the impact of Covid-19 on WCT, particularly by the students. Despite this, the direct impact of Covid-19 on desirable preparedness traits appeared to be minimal, and there is still applicability of the data to real-world settings post-pandemic.

Where interviews are held is important [63]. Hosting the group interviews online, where participants took part from their own homes, is likely to have facilitated participant comfort and a welcoming, nonthreatening, and neutral environment. Additionally, there was a reduced influence of non-verbal communication on the group dynamics; participants may have been more forthcoming but also found it difficult to indicate when they wanted to talk. This, combined with reduced control of the moderator on a virtual platform, may explain why a couple of participants dominated the conversation in their group interviews. Fortunately, no technical problems or confidentiality issues were experienced, which are additional considerations when interviewing online.

A limitation of the data analysis was that solicited (talk in response to direct questions) and unsolicited (through the sharing experiences and stories) conceptualisations of preparedness were not compared as it has been in other preparedness research [36]. Identifying conscious and therefore dominant conceptualisations of preparedness may have given insights into which are more important to participants.

Recruiting some stakeholder groups from a single institution (namely, final year students, recent alumni, and faculty) may limit the generalisability of the findings. Additionally, the definition of workplace clinical training provided to interview participants did not include longitudinal or pre-clinical workplace learning, such as extra-mural studies in the UK. It is possible, and likely, that preparedness for these types of experiences may be different to that described here.

## Conclusion

This study has demonstrated that preparedness for veterinary WCT is complex and highly contextualised. Whilst acknowledging that the transition into veterinary WCT is likely to be a CILP and students can never be fully prepared for it, using our conceptual framework (the “Preparedness Toolkit” [42]) as a starting point, we have inductively identified preparedness characteristics that would be beneficial for students to possess to facilitate their learning and working in the veterinary workplace. Future work should assess the relative importance of these preparedness characteristics and to determine whether there are differences in perspectives between stakeholder groups.

## Methods

### Study design

Group interviews are based on the schema of a one-to-one semi-structured interview but duplicated with multiple participants to address a particular research question [64]. Group interviews were selected for this study because of an interest in exploring individual, multiple realities of preparedness and the lived experience of participants (the emic perspective), but were not overly concerned in making meaning of the interactions between participants, which distinguishes them from focus groups [65].

It is likely that perceptions of preparedness for WCT are dependent on what an individual believes to be necessary for students’ upcoming experiences. This will be highly influenced by their own personal experiences and context. If preparedness is socially and experientially bound, and somewhat personalised, it is appropriate to take a constructivist approach to this research. Ontologically, constructivists believe that multiple ‘truths’ can exist and epistemologically that “we are shaped by our lived experiences, and these will always come out in the knowledge we generate as researchers and in the data generated by our subjects” [66] (pg.117).

### The “Preparedness Toolkit” conceptual framework

The “Preparedness Toolkit” conceptual framework [42] guides both the data collection and initial stages of analysis of this study. The tools within it are categorised into tool-types: students’ personal attributes, knowledge and skills, behaviours and awarenesses (Fig. 1).

### Participants and sampling

To obtain a holistic view of preparedness, a range of stakeholder groups were identified by the research team: clinical supervisors, academic educationalists, veterinary school faculty, final year veterinary students and recent student alumni. Inclusion criteria for these groups were made available in the recruitment material sent to the relevant groups, and fulfilment of certain criteria could be verified through the demographic survey sent to all participants within 24 h of the interview. Inclusion criteria are presented in Appendix 1.

Purposive and convenience sampling was used. Final year students, recent student alumni and faculty participants were recruited from the researchers’ home university (University of Surrey). Clinical supervisors and academic educationalists were recruited from across the UK.

In response to restrictions resulting from the Covid-19 pandemic, group interviews were held online, over a two-month period. The optimal number of participants for an online focus group has been identified as three to five [67, 68]; this limits synchronisation problems, and allows participants to see each other on split screens. Interview organisation was based on these numbers. All participants in each interview were of the same stakeholder group (e.g., only students or only faulty) to maximise participant comfort and the co-construction of knowledge based on similar experiences.

### Group interview procedure

Group interviews were held and recorded using online conferencing software (Zoom©^1^). The virtual room was opened fifteen minutes prior to the scheduled start time to identify any technical difficulties and to promote participant interaction. Participants were given a short introductory presentation on the motivation for the study and were presented with the “Preparedness Toolkit” [42] as a point of departure for the upcoming interview.

To gain an emic perspective, open questions from a prompt sheet that aligned with the “Preparedness Toolkit” framework [42] were used, followed by closed questions to probe participants. New lines of enquiry were generated from the previous participant talk. Participant validation (‘member checking’) was performed using intermittent follow up questions such as “Can I check that what you mean is…?”, followed by an affirmative answer from the participant. Prior to moving on to a new broad topic, participants were asked if they had anything else to add.

Although data saturation is not always valued in the constructivist paradigm, data sufficiency [69, 70] (‘good enough’) was considered important in order to achieve a rich perspective on preparedness. Each group interview was scheduled to last approximately one hour, but interviewees were invited to stay longer if further discussion points came to light. Data sufficiency was partly determined by concluding all interviews with the question “anything else?”, until no more suggestions were made. In a single instance it was felt that this had not been achieved in a reasonable timeframe and specific participants were invited to return for a second session.

Intelligent verbatim transcription by a professional transcriber^2^ produced anonymised text transcripts from the audio files.

### Template analysis

**Fig. 2:**
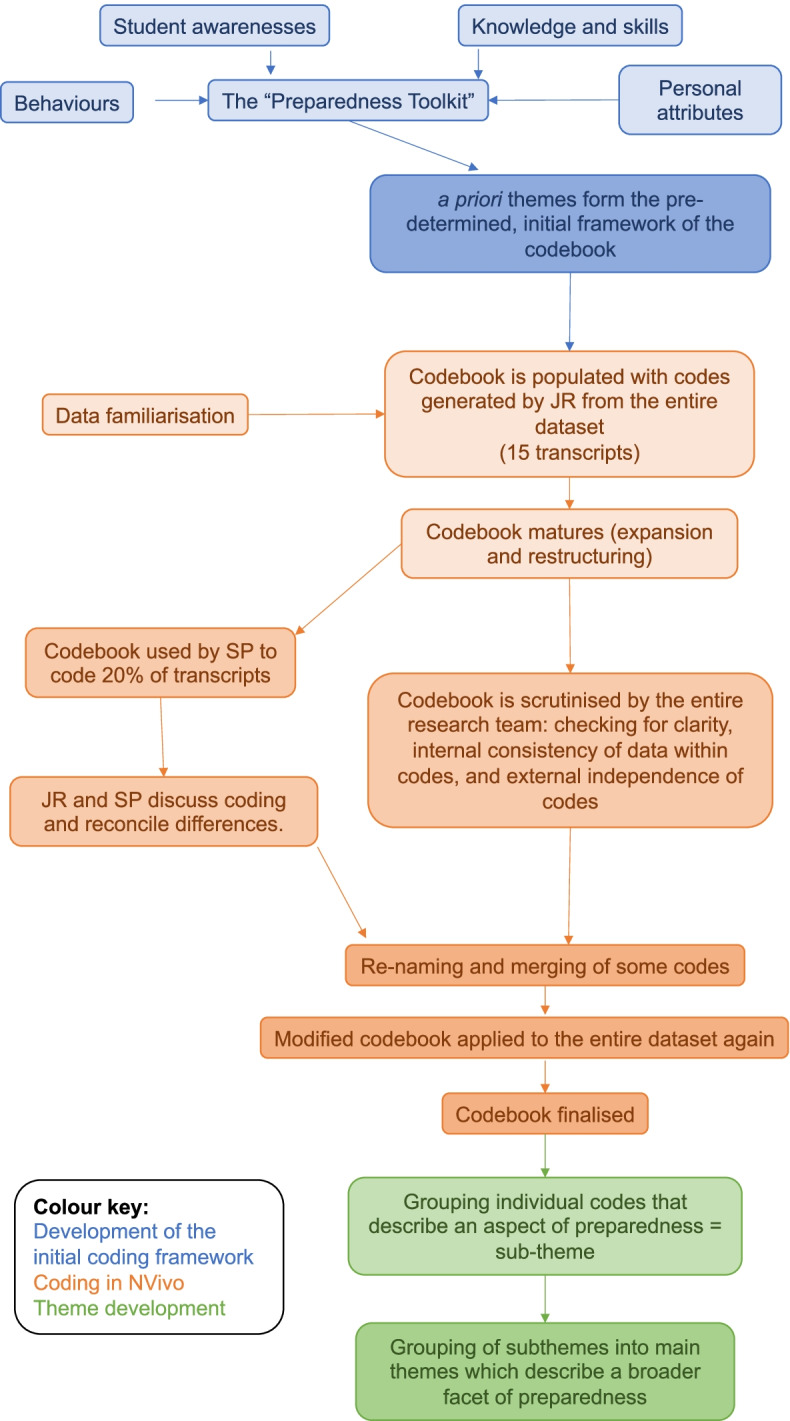
The process of thematic analysis using a template analysis approach

The analysis of the text transcripts was informed by King’s template analysis [71, 72] (Fig. 2). Template analysis is a flexible form of the codebook approach to thematic analysis [73–75]. This approach was chosen because it combines the values of researcher subjectivity and contextualised knowledge, with a structured approach to coding [74]. Additionally, template analysis can use a priori themes to produce a pre-determined framework for coding in the initial stages [71, 72]. In this case, the tool-types from the “Preparedness Toolkit” [42] were used to populate the preliminary framework. In template analysis, the codebook is dynamic and further codes and/or themes are generated inductively throughout the coding process.

Analysis was performed by the lead researcher (JR) using NVivo.^3^ Firstly, data familiarisation was performed during the collection phase through detailed notetaking, followed by reading through the transcripts whilst listening to the audio files for transcription accuracy. Initial codes were generated in the second stage, these were identified as the most granular but independent elements of the data set, which fit rationally into the pre-determined framework and described a single preparedness characteristic e.g., “knowledge of anatomy”. The codebook grew and developed as analysis through the transcripts progressed. By the last two transcripts, only a single code was added to the codebook; this provided further evidence for the achievement of data sufficiency.

After the initial coding round, the codebook was sent to members of the supervisory team (KJ, PC and SP) and 20% of the transcripts were sent to one supervisor (SP). This sample of transcripts was coded by SP using the codebook. SP and JR discussed and reconciled differences in their coding of that sample of transcripts. Although inter-rater reliability is not considered important in the codebook approach because coding is regarded as inherently subjective [75], this exercise was useful for improving the clarity of the framework. All supervisors scrutinised the codebook for internal consistency and external independency of the codes resulting in some codes being merged or re-named. An audit trail was compiled by keeping written notes on the decisions made as the codebook was modified and the justification for those decisions.

The codebook was applied to the entire dataset again to operate those changes. No new codes were added at this point indicating that thematic saturation [76] had been achieved. The final codebook had a hierarchical structure which is consistent with the template analysis approach [71].

Themes were conceptualised as topic summaries [73] and were actively identified. All individual codes were printed onto tickets of paper and were arranged into groups that were frequently talked about together or collectively explained an interesting aspect of preparedness. These groups of codes were designated as sub-themes and given names. In parallel, the encapsulated data were checked for consistency with the code and subtheme names that enveloped them. Candidate quotes were highlighted in the text which captured each sub-theme concisely to provide support for their existence. Sub-themes themselves were grouped into main themes, which explained a broader facet of preparedness, and were named.

### Positionality statement for data analysis

Given that this research was performed within the constructivist philosophical paradigm, it is important to clarify the exact position from which the researchers have approached the work. The lead researcher (JR) who facilitated and analysed the group interviews was a six-year graduated veterinary surgeon. They have experienced WCT personally as an undergraduate, supervised veterinary students in practice and now work within a veterinary school faculty. Positioned in the “middle ground”, as a neutral but informed researcher, they were able to build rapport with both staff and students which facilitated disclosure and authenticity.

From an epistemological standpoint, it is likely that the data produced from the group interviews was, at least partially, socially constructed and influenced by the facilitator’s experiences and interactions with the participants, as is consistent with a constructivist approach. However, no distorting effects on the authenticity of responses were appreciated and there were no appreciable in-group hierarchies, vulnerable participants, or secondary participant motives present to our knowledge.

## Supplementary Information

**Additional file 1: Appendix 1.** Participant inclusion criteria.

## Data Availability

The datasets generated and analysed in this study are not publicly available because they contain personal data. Although interview participants have been de-identified in the text transcripts, the data is highly contextualised and should be considered pseudo, not fully, anonymous. In other words, with additional information, there is a possibility of participants being identified. Participants did not consent to the use of data in publication where results could be personally identifiable. Therefore, to protect the anonymity of the participants, the audio files and text transcripts are not available, however representative quotes are provided in the results section, which are not personally identifiable.

## Abbreviations

CILP: Critically intensive learning period
RCVS: Royal College of Veterinary Surgeons
WCT: Workplace clinical training

## Footnotes

1 Zoom Video Communications©. 55 Almaden Boulevard, Suite 400, 500, 600, San Jose, CA 95,113.

2 TP Transcription Ltd. Pen y Banc, Ystrad Pont, Denbigh, Wales, LL16 4RW.

3 NVivo 12©. QSR International Ltd©. Tower 2, Level 2, Chadstone Shopping Center, 1341 Dandenong Road, Chadstone, Victoria, 3148, Australia.
