# Supplementary material for: Stakeholder perspectives on veterinary student preparedness for workplace clinical training – a qualitative study
Source: BMC Vet Res. 2022 Sep 9;18:340. doi: 10.1186/s12917-022-03439-6 (PMC9461096; doi:10.1186/s12917-022-03439-6)
Supplement: Supplementary file 1 — Additional file 1: Appendix 1. Participant inclusion criteria. [file 12917_2022_3439_MOESM1_ESM.pdf]

Appendix 1 – participant inclusion criteria

Clinical supervisor inclusion criteria were a veterinary surgeon with content expertise who is appropriately trained to be responsible for overseeing a specified veterinary student's work during WCT and providing constructive feedback with respect to the learning outcomes of the programme (examples might include staff clinicians, residents, and interns).

Veterinary school faculty inclusion criteria were those members of the University of Surrey veterinary school academic staff who are directly involved in preparing students for WCT (e.g. Veterinary Clinical Training Fellows or those teaching a clinical subject or skills), and have personal experience of WCT as a student themselves or as a veterinary surgeon in practice supervising students.

Academic educationalist inclusion criteria were UK based, graduated as a veterinary surgeon (although they did not have to be a practising member of the RCVS), and hold one of the following: Master's degree (MSc or MRes), PhD or EdD in medical or veterinary education, or hold a Head of/ Chief of/ Director of/ Professorship in Veterinary Education or Clinical Education, or be a Principal Fellow of the Higher Education Academy, or be a member of the Veterinary Schools Council Education Committee, and their email address must be available in the public domain.

Student inclusion criteria were that the individual must be in their final year of the University of Surrey veterinary medicine degree programme and taking their WCT at the time of the interview.

Recent alumni inclusion criteria were that the individual must have graduated from the University of Surrey veterinary medicine degree programme in the last two years, having taken part in WCT. For convenience, the alumni reps (graduates who had volunteered their contact details for follow up by the University) were approached and recruited.
